# Patient-Specific Restoration of Constitutional Alignment Within Predefined Safety Boundaries Using Three-Dimensional Navigation in Primary Total Knee Arthroplasty: One-Year Clinical and Radiographic Outcomes

**DOI:** 10.3390/jcm15062441

**Published:** 2026-03-23

**Authors:** Maximilian F. Kasparek, Tobias Scheidl, Oliver Haider, Gyula Kiss, Anna Jungwirth-Weinberger, Maximilian Muellner, Valerie Ladstaetter, Thomas Muellner

**Affiliations:** 1 Department of Orthopedic Surgery and Traumatology, Evangelisches Krankenhaus, Hans-Sachs-Gasse 10-12, 1180 Vienna, Austria; 2 Medical University of Vienna, Vienna General Hospital, Waehringer Guertel 18-20, 1090 Vienna, Austria; 3 Center for Musculoskeletal Surgery, Charité—Universitätsmedizin Berlin, Charitéplatz 1, 10117 Berlin, Germany

**Keywords:** total knee arthroplasty, functional alignment, kinematic alignment, 3D navigation, knee osteoarthritis

## Abstract

**Background/Objectives**: This study investigates a surgical concept that restores constitutional bony alignment within predefined safety boundaries in primary total knee arthroplasty (TKA) using modern 3D navigation. The technique combines a standard knee implant with advanced navigation technology to achieve patient-specific alignment and recreate native joint mechanics. One-year outcome was evaluated to assess first clinical results. **Methods**: In this retrospective study, a consecutive series of 185 TKAs (171 patients) was analyzed. All patients underwent patient-specific restoration of constitutional alignment within predefined safety boundaries using a 3D navigation system and a standard knee arthroplasty implant. The clinical outcomes were assessed using the 2011 Knee Society Score (KSS), the Forgotten Joint Score (FJS-12), the UCLA Activity Scale, and a five-step Likert scale to evaluate satisfaction. **Results**: In a total of 87.6% of cases, the patients reported being either satisfied or very satisfied with their TKA. No patients reported strong dissatisfaction. The KSS demonstrated significant improvements in all subcategories (all *p* < 0.001). The FJS-12 increased significantly from a preoperative average of 32.5 points to 79.3 points postoperatively (*p* < 0.001). The mean UCLA activity score rose from 4.9 preoperatively to 6.6 postoperatively (*p* < 0.001). In 97.7% and 90.2% of cases, the femoral mechanical angle (FMA) and tibial mechanical angle (TMA) bone cuts were within ± 1° of the planned angles. A strong correlation was observed between the planned and verified bone cuts for the FMA (ρ = 0.939) and the TMA (ρ = 0.875). **Conclusions**: Patient-specific restoration of constitutional alignment within predefined safety boundaries in primary TKA using modern 3D navigation is a promising strategy for personalized joint reconstruction using a standard knee arthroplasty implant. It combines precision and reproducibility with high patient satisfaction by respecting each patient’s constitutional alignment.

## 1. Introduction

Total knee arthroplasty (TKA) remains the standard treatment for end-stage knee osteoarthritis [1]. Mechanical alignment (MA), aiming for a neutral hip–knee–ankle angle (HKA = 0° ± 3°), has long been considered the gold standard to ensure even load distribution and long-term implant survival [2,3,4]. Nevertheless, about 20% of patients remain dissatisfied after TKA [5,6], partly due to the neglect of individual anatomical variations.

Functional alignment (FA) is a patient-specific, balance-driven approach that aims to restore the native joint line while achieving stable and symmetrical extension and flexion gaps [7]. Rather than strictly following a neutral mechanical axis, FA relies on intraoperative feedback, typically with navigation or robotic assistance, to guide component positioning within predefined safe limits. The objective is to minimize soft tissue releases and promote more natural knee function. However, since a consistent definition of the various combinations and techniques used within FA is currently lacking, the development of a more detailed and standardized nomenclature is required [8].

Kinematic alignment (KA) is a surgical technique that aims to restore the pre-arthritic joint line and native knee kinematics by positioning prosthetic components to match the patient’s individual anatomy [9]. Unlike mechanical alignment, KA does not impose strict limits on the hip–knee–ankle (HKA) angle or joint line obliquity, thereby allowing fully individualized alignment based on each patient’s pre-arthritic anatomy. Restricted kinematic alignment (rKA) follows the same patient-specific restoration principles as KA but constrains component positioning within predefined safety boundaries to avoid excessive varus or valgus alignment [9].

Constitutional alignment refers to a patient’s individual pre-arthritic coronal limb alignment, acknowledging that many individuals naturally exhibit constitutional varus or valgus rather than neutral mechanical alignment [10]. The present concept incorporates constitutional bony alignment as a reference within a functional alignment workflow. Using three-dimensional (3D) navigation, this approach aims to optimize soft tissue balance throughout the full range of motion while maintaining alignment within predefined safety limits.

The current study evaluates the short-term outcomes of a surgical concept that applies FA based on constitutional bony alignment using modern three-dimensional (3D) navigation. This technique employs a standard knee arthroplasty implant in combination with advanced navigation, with the aim of restoring patient-specific alignment and reconstructing native joint mechanics.

## 2. Materials and Methods

This study analyses a consecutive series of 204 patients (219 TKAs) who underwent navigated primary TKA using the LEGION^®^ implant system (Smith & Nephew, Memphis, TN, USA). The surgical procedures were performed between September 2021 and March 2023 by two experienced arthroplasty surgeons at a single center. The KNEE3^®^ 3D Navigation System (Brainlab, Munich, Germany) was utilized in conjunction with a FA philosophy, aiming to restore each patient’s constitutional anatomy. Inclusion criteria comprised ambulatory patients with primary knee osteoarthritis, defined as individuals capable of independent ambulation who were neither permanently bedridden nor wheelchair dependent. No restrictions were applied regarding the degree of coronal limb deformity.

Exclusion criteria included patients with secondary osteoarthritis due to prior infection or post-traumatic arthrosis, as well as those with a history of high tibial osteotomy (HTO). Furthermore, patients with periprosthetic fractures or periprosthetic joint infection were excluded from the final analysis.

The current study was conducted after the approval of the institutional ethics committee.

**Figure 1:**
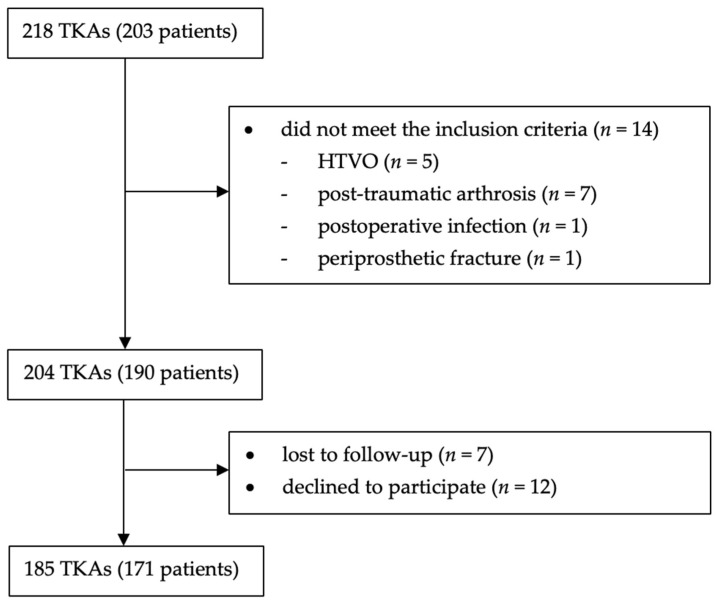
Diagram presenting all enrolled patients.

A total of 218 TKAs (203 patients) were identified. As shown in Figure 1, 14 cases did not meet the previously mentioned inclusion criteria. Of the remaining 204 TKAs (190 patients), seven were lost to follow-up and 12 declined to participate. Thus, 185 TKAs (171 patients) were available for analysis.

### 2.1. Clinical Outcomes

The 2011 KSS [11] was used to evaluate objective knee function, symptoms, patient satisfaction, patient expectations, and overall functional status. The FJS-12 [12] was used as an indicator of how often a patient’s knee joint was perceived as painful or uncomfortable in daily activities, with higher scores indicating less awareness of the joint. The UCLA Activity Score [13] was used to assess both current and desired levels of physical activity, similar to the approach previously described by Scheidl et al. [14], in order to further evaluate the fulfillment of patients’ activity expectations. The score ranges from 1 (bedridden) to 10 (regular participation in high-impact sports).

The patients’ overall satisfaction with their TKA was documented using a Likert scale, which comprised five possible responses, ranging from “very satisfied” and “satisfied”, to “neutral”, “dissatisfied” and “very dissatisfied.” Furthermore, patients were asked to provide feedback on their overall condition and to report any adverse events that occurred during the follow-up period. Preoperative data was collected on the day before surgery.

### 2.2. Radiological Assessment

Standardized preoperative and postoperative radiographs, including full-length weight-bearing, anteroposterior, lateral, and patellar tangential views, taken the day before and three days after surgery, were reviewed by two independent readers. The measured parameters included the patient’s hip–knee–ankle angle (HKA), tibial mechanical angle (TMA), femoral mechanical angle (FMA), tibial slope, joint line, patella shift and patella tilt angle. All parameters were measured preoperatively and postoperatively as previously described [15,16].

The joint line was measured using the adductor tubercle to joint line distance, as previously reported by Hofmann et al. [17]. Postoperative radiographs were also reviewed for signs of implant loosening.

### 2.3. Surgical Technique

Prior to the surgery, FMA, TMA and HKA were measured, and phenotypes were classified according to the CPAK classification [18]. In each case, the standard LEGION^®^ implant system (Smith & Nephew, Memphis, TN, USA) was used in combination with the Brainlab KNEE3^®^ navigation system (Brainlab, Munich, Germany). This involved the use of an optical infrared camera, which communicates with a femoral and tibial tracker, thereby obviating the necessity for preoperative MRI or CT imaging.

Anesthesia was either general or spinal, based on the patient’s comorbidities or preferences. The patients were positioned supine with 90° knee flexion and prophylactic antibiotics were administered. All patients underwent a midline skin incision and a midvastus approach. The navigation trackers were affixed to the femur and tibia and measurements for leg axis, range of motion (ROM), and medio-lateral ligament balance, across the full ROM, were recorded.

Resection planning was performed intraoperatively within predefined safety boundaries. These boundaries were established to maintain alignment within defined safety limits while allowing restoration of each patient’s constitutional bony anatomy. The safety boundaries were defined as follows: a FMA and TMA between 86° and 94° (corresponding to ±4° from neutral mechanical alignment), and a HKA between 175° and 183°, in accordance with the alignment ranges previously described by Bonnin et al. [19]. All bony resections were planned within these limits; no bone cuts were performed outside these predefined safety boundaries. Bone resections were continuously monitored intraoperatively using the 3D navigation system to verify accuracy and adherence to the planned alignment.

The distal femoral cut was performed first (femur first technique), followed by the proximal tibial resection. After performing the resections, the mediolateral soft tissue balance was checked with a tension measurement and a spacer to assess the extension gap. Femoral rotation was determined following establishment of the extension gap, based on the optimal intraoperative ligament balance, within a predefined safety range from 3° of internal to 6° of external rotation. Posterior osteophytes were routinely removed. If the posterior cruciate ligament (PCL) was intact, a cruciate retaining (CR) implant was used.

In cases of PCL insufficiency, posterior-stabilized (PS) implants with a patella replacement were used. Trial components were used to assess optimal polyethylene inlay (HXLPE) height, leg alignment, and ligament tension. If medial or lateral soft tissue releases were required, they were performed. Patellar osteophyte removal or lateral facetectomy was performed as necessary. In cases of advanced patellar osteoarthritis or deep trochlear, the patella was resurfaced. Following the implementation of the final components, local infiltration anesthesia was applied intraoperatively, and the wound was closed without the use of a drainage. The skin was closed using an intracutaneous suture. A modern plaster dressing was applied, and patients were mobilized with a period of four to six weeks of crutches.

## 3. Statistical Analysis

Statistical analyses were performed using IBM SPSS Statistics (version 29.0.2.0; IBM, Armonk, NY, USA). Normality was assessed with the Shapiro–Wilk test. Depending on data distribution, paired *t*-tests or Wilcoxon signed-rank tests were applied for preoperative and postoperative comparisons, and the Mann–Whitney U test for subgroup analyses. Spearman’s rank correlation coefficient (ρ) with corresponding 95% confidence intervals was calculated to assess the association between planned and verified TMA/FMA. A two-tailed *p*-value < 0.05 was considered significant. Intraobserver and interobserver reliability showed excellent agreement (ICC = 0.94).

## 4. Results

The cohort consisted of 103 women (60.2%) and 68 men (39.8%), with a mean age of 74.7 ± 9.2 years and BMI of 27 ± 4.78 kg/m2. In total, 101 right and 84 left knees were operated on, including 14 bilateral cases. All patients received the LEGION^®^ implant system (Smith & Nephew, Memphis, TN, USA); 162 cases (87.6%) used a cruciate-retaining (CR) and 23 (12.4%) a posterior-stabilized (PS) design, including eight constrained inlays.

In a total of six cases (3.2%) a soft tissue release was performed, comprising two medial and three lateral releases, as well as one posterior capsule release. The mean follow-up was 15.2 ± 4.3 months.

Overall, 162 patients (87.6%) were satisfied or very satisfied, while 18 patients (9.7%) were neutral and five patients (2.7%) were dissatisfied. No patients reported strong dissatisfaction.

The 2011 KSS showed significant improvement across all subgroups. The FJS-12 improved from 32.5 ± 14.9 to 79.3 ± 17.3, and the UCLA score from 4.9 to 6.6 (*p* < 0.001). No significant differences were observed between varus and valgus subgroups. Details can be found in Table 1.

The planned tibial slope was accurately restored from a mean of 8.3° preoperatively to 3.5° postoperatively, including 4° of inlay slope. The joint line was preserved within ±4 mm in 97.8% of cases. Patellar alignment improved significantly, with normal patellar shift increasing from 60.9% preoperatively to 84.7% postoperatively (*p* < 0.001), and normal patellar tilt from 60.3% to 75.1% (*p* = 0.015) [20]. Detailed radiographic parameters are summarized in Table 2.

Bone resections showed high accuracy, with over 97% of femoral and tibial cuts within ±1° of the planned values. Strong correlations were observed between planned and verified angles for both FMA (ρ = 0.94; 95% CI, 0.91–0.96; *p* < 0.001) and TMA (ρ = 0.88; 95% CI, 0.83–0.91; *p* < 0.001). Details are provided in Table 3.

Preoperative and postoperative anatomical phenotypes were classified according to the coronal plane alignment of the knee (CPAK) classification [18] based on FMA, TMA, and HKA measurements. Overall, 35% of knees maintained their preoperative CPAK type, while 65% changed postoperatively. Similar distribution patterns were observed for varus and valgus subgroups.

The most common preoperative phenotypes were CPAK types 1–3, with a postoperative shift mainly toward type two and five. Detailed distributions are provided in Table 4.

When comparing cases with unchanged versus changed CPAK type, no significant differences were found in postoperative KSS, FJS-12, or UCLA scores, except for a minor variation in the desired UCLA score (*p* = 0.044).

Two patients developed a suture granuloma (foreign body granuloma caused by suture material), and one patient experienced delayed wound healing. All cases were treated conservatively. One patient had transient pain at the tracker sites, and one patient developed recurrent knee swelling requiring subsequent radiosynoviorthesis. Furthermore, two patients (1.1%) required secondary patellar resurfacing.

## 5. Discussion

The most important finding of this study is that reconstructing constitutional alignment within predefined safety boundaries using 3D navigation in primary TKA is a reproducible technique that requires minimal soft tissue release. Clinical data showed significant improvements in pain, function, and activity levels. Radiographic assessments confirmed that most knees were aligned within the planned target zone. Patellar alignment improved substantially in terms of both tilt and shift, though two patients required secondary patellar resurfacing.

The results of the current study are comparable to recent studies on FA and rKA. Hazratwala et al. [21] reported a consecutive series of 165 TKAs performed using navigated FA. They demonstrated reproducible results with high patient satisfaction and required soft tissue releases in 14.5% of cases. Similar findings were observed in the present study, indicating that functional alignment using 3D navigation reduces the need for soft tissue releases.

When comparing FA to MA, the recent literature suggests improved patient-reported outcomes with FA, particularly regarding soft tissue balance and early functional recovery. Young et al. [22] recently presented a randomized controlled trial comparing MA and FA, confirming that FA required significantly fewer soft tissue releases while delivering at least comparable clinical outcomes. However, long-term superiority has not been consistently demonstrated. Sangaletti et al. [23] reported comparable outcomes at five years of follow-up. These findings suggest that alignment philosophy alone may not determine clinical outcomes; rather, the precision and reproducibility of the chosen technique appear to be critical factors.

Gregori et al. [24] specifically examined FA in robotic-assisted TKA for valgus deformity. They observed excellent short-term outcomes, a 1.7% complication rate, and 86% of patients within the safe coronal alignment zone (HKA 177–183°). Their reported improvements in KSS, Oxford Knee Score (OKS), and FJS-12 are comparable to the results of the current study, suggesting that individualized alignment strategies can be applied safely even in challenging deformities.

The results of this study are comparable to the current literature, demonstrating high patient satisfaction and high Forgotten Joint Scores one year postoperatively. In the current study, the 2011 KSS and FJS-12 both demonstrated significant improvements postoperatively. This matches reports by Matsumoto and Kuroda et al. [25,26,27] and Chompoosang [28], who likewise found higher functional outcomes with KA or FA approaches compared to MA. The high FJS-12 scores observed in the present study reflect low joint awareness in everyday activities. Comparable FJS-12 values have been reported by Gousopoulos et al. [29] in a CT-based custom TKA cohort, further validating that individualized alignment—whether via navigation or patient-specific custom-made implant—yields superior functional outcomes. The preoperative expectations and postoperative UCLA activity scores in the present cohort were closely aligned, indicating that patients largely achieved their anticipated activity levels.

Restoration of alignment was achieved with high precision. Eighty-five percent of knees were aligned within the predefined safe zone (FMA and TMA 85–95°, HKA 175–183°). These findings closely resemble those of Bonnin et al. [19], who showed that 84% of custom TKAs were aligned within target zones. Custom-made TKA combined with personalized alignment has been shown to achieve high satisfaction rates and strong PROMs, as reported by Ratano et al. [30] and Gousopoulos et al. [29].

While custom implants may provide enhanced reproduction of patient-specific anatomy, they require advanced imaging and offer reduced intraoperative flexibility, as adaptation of the surgical plan is not possible due to the use of prefabricated cutting blocks. In contrast, the use of a standard implant combined with navigation allows individualized alignment within defined safety boundaries while preserving intraoperative adaptability. Nevertheless, standard implants may not fully replicate patient-specific anatomical variations, which may represent a limitation compared to custom solutions. However, our results demonstrate that excellent outcomes can be achieved using conventional implants with navigated FA, while maintaining the intraoperative option to adjust resection parameters and implant type (CR, PS, or constrained designs).

FA with restoration of the constitutional alignment using 3D navigation also has good results concerning patellar parameters. A significant correction of patellar tilt and shift was achieved, in line with biomechanical data from Koh et al. [31] and Terashima et al. [32], who demonstrated that KA and external femoral component rotation reduce patellofemoral stress and improve tracking. Proper patellar restoration is essential to avoid anterior knee pain and long-term complications.

One of the advantages of FA and rKA strategies is the reduced need for soft tissue release. In the current study, only a minority of patients required additional soft tissue releases. This finding parallels Clapp et al. [33], who demonstrated that robotic-assisted FA required significantly fewer releases than both conventional and accelerometer-based navigation techniques. Minimizing releases preserves natural knee kinematics and likely contributes to better early pain scores and faster recovery.

Recent work has emphasized the importance of CPAK classification. Our study demonstrated reduced postoperative variation across this classification, reinforcing that navigated FA is reproducible. However, postoperative changes in CPAK classification were observed in 65% of knees and were not associated with inferior clinical outcomes. This suggests that exact phenotype restoration may be less important than achieving alignment within a predefined functional safe zone. This aligns with the findings of Bertugli et al. [34], who showed that changes in CPAK class after robotic-assisted FA did not negatively affect outcomes, suggesting that restoration within a functional safe zone is more important than exact phenotype matching. Similarly, Yang et al. [35] emphasized that restoring joint line obliquity (JLO) was more predictive of outcomes than overall limb alignment, highlighting the value of individualized approaches.

Several limitations must be acknowledged. First, this was a retrospective analysis without a direct comparison group, which restricts external validity. Second, the mean follow-up of 15 months does not permit conclusions regarding long-term implant survivorship or the occurrence of late complications. Third, patient-reported outcome measures (PROMs) are inherently subjective. Fourth, both cruciate-retaining and posterior-stabilized implant designs were used. Different implant selection may influence kinematics and functional outcomes. Finally, the study was conducted during the learning curve of adopting navigated FA, though outcomes were consistently favorable.

Long-term randomized controlled trials are required to determine whether FA and rKA strategies improve implant survival compared to MA. Further research should also examine subgroup-specific benefits, such as different deformity types or different CPAK classes. Integration of navigation and robotic systems with patient-specific planning could eventually optimize both accuracy and efficiency, though cost-effectiveness remains a concern.

## 6. Conclusions

The reconstruction of constitutional alignment within predefined safety boundaries using 3D navigation in primary TKA is a reproducible technique demonstrating high radiographic accuracy and favorable short-term clinical outcomes with high patient satisfaction. This technique enables individualized alignment within predefined safety boundaries while utilizing standard implant designs. Nevertheless, longer-term follow-up and comparative studies are needed to determine whether this approach offers advantages over established alignment strategies.

## Figures and Tables

**Table 1:** Clinical data for the subgroups of KSS, FJS-12, and UCLA score.

		Mean	*Range*	*SD*	*p*-Value
**Objective Knee Score total** **(max. 100 points)**	preoperative	**70.7**	*38 to 95*	*11*	<0.001 *
	postoperative	**94.8**	*80 to 100*	*4.4*	
**Symptoms** **(max. 25 points)**	preoperative	**11.6**	*0 to 24*	*4.4*	<0.001 *
	postoperative	**22.1**	*12 to 25*	*3*	
**Patient Satisfaction** **(max. 40 points)**	preoperative	**17.7**	*4 to 36*	*6.4*	<0.001 *
	postoperative	**34.7**	*16 to 40*	*5.4*	
**Patient Expectations** **(max. 15 points)**	preoperative	**14**	*3 to 15*	*1.8*	<0.001 *
	postoperative	**10**	*3 to 15*	*2.6*	
**Functional Knee Score total** **(max. 100 points)**	preoperative	**47.8**	*4 to 93*	*15.5*	<0.001 *
	postoperative	**80.4**	*39 to 100*	*12*	
**Forgotten Joint Score (FJS-12)** **(max. 100 points)**	preoperative	**32.5**	*0 to 71*	*14.9*	<0.001 *
	postoperative	**79.3**	*0 to 100*	*17.3*	
**Desired UCLA Score** **(max. 10 points)**	preoperative	**6.7**	*2 to 10*	*1.65*	<0.001 *
	postoperative	**6.9**	*3 to 10*	*1.62*	
**Current UCLA Score** **(max. 10 points)**	preoperative	**4.9**	*2 to 10*	*2.17*	<0.001 *
	postoperative	**6.6**	*2 to 10*	*1.77*	

* Wilcoxon signed-rank test. SD = standard deviation.

**Table 2:** Preoperative and postoperative measurements of HKA, FMA, TMA, tibial slope, joint line, patella shift and patella tilt angle.

	Mean	*Range*	SD	*p*-Value
**Pre HKA, in °**	180.5	*167.1 to 196.8*	*4.45*	0.098 *
**Post HKA**	180.1	*173.9 to 191.3*	*2.68*	
**Pre FMA, in °**	92.7	*83.6 to 100.8*	*2.76*	**<0.001** †
**Post FMA**	91.6	*87 to 95.6*	*1.78*	
**Pre TMA, in °**	87.9	*78.2 to 98.1*	*3.13*	**<0.001** †
**Post TMA**	88.5	*84.4 to 96.4*	*1.87*	
**Pre tibial slope, in °**	8.3	*1.7 to 15*	*2.82*	**<0.001** †
**Post tibial slope**	3.5	*0 to 9.4*	*1.9*	
**Pre joint line, in mm**	43.9	*32.9 to 57.7*	*4.86*	0.514 *
**Post joint line**	44	*32.1 to 57.8*	*5.1*	
**Pre patella shift, in mm**	1.9	*0 to 11*	*2.32*	**<0.001** †
**Post patella shift**	1	*0 to 9*	*1.92*	
**Pre patella tilt angle, in °**	4.5	*−4.5 to 17.4*	*3.92*	**0.015** †
**Post patella tilt angle**	3.5	*−3.8 to 14.3*	*2.81*	

* Paired *t*-test. † Wilcoxon signed-rank test. SD = standard deviation.

**Table 3:** Comparison of planned and verified bone cuts for FMA and TMA.

Parameters	Planned			Verified		
	Mean	Range	SD	Mean	Range	SD
**FMA**	1.25	−3 to 4	1.49	1.24	−4 to 3.5	1.61
**TMA**	−1.72	−4 to 3	1.5	−1.88	−5 to 3	1.62

**Table 4:** Preoperative and postoperative phenotypes according to CPAK classification (MacDessi et al. [18]).

Pre OP CPAK	Frequency (*n*)	Percentage (%)	Post OP CPAK	Frequency (*n*)	Percentage (%)
1	42	23.3	1	18	10.0
2	39	21.7	2	56	31.1
3	44	24.4	3	21	11.7
4	13	7.2	4	23	12.8
5	16	8.9	5	39	21.7
6	23	12.8	6	23	12.8
7	2	1.1	7	0	0
8	1	0.6	8	0	0
9	0	0	9	0	0
CPAK maintained pre and post OP (%)					
Yes			No		
35.0			65.0		

## Data Availability

The original contributions presented in this study are included in the article. Further inquiries can be directed to the corresponding author.
